# Optimization of Corneal Epithelial Progenitor Cell Growth on* Bombyx mori* Silk Fibroin Membranes

**DOI:** 10.1155/2016/8310127

**Published:** 2016-08-28

**Authors:** Thomas A. Hogerheyde, Shuko Suzuki, Jennifer Walshe, Laura J. Bray, Sally A. Stephenson, Damien G. Harkin, Neil A. Richardson

**Affiliations:** ^1^School of Biomedical Sciences, Faculty of Health and Institute of Health & Biomedical Innovation, Queensland University of Technology, 2 George Street, Brisbane, QLD 4001, Australia; ^2^Queensland Eye Institute, 140 Melbourne Street, South Brisbane, QLD 4101, Australia

## Abstract

Scaffolds prepared from silk fibroin derived from cocoons of the domesticated silkworm moth* Bombyx mori* have demonstrated potential to support the attachment and growth of human limbal epithelial (HLE) cells* in vitro*. In this study, we attempted to further optimize protocols to promote the expansion of HLE cells on* B. mori* silk fibroin- (BMSF-) based scaffolds. BMSF films were initially coated with different extracellular matrix proteins and then analysed for their impact on corneal epithelial cell adhesion, cell morphology, and culture confluency. Results showed that collagen I, collagen III, and collagen IV consistently improved HCE-T cell adherence, promoted an elongated cell morphology, and increased culture confluency. By contrast, ECM coating had no significant effect on the performance of primary HLE cells cultured on BMSF films. In the second part of this study, primary HLE cells were grown on BMSF films in the presence of medium (SHEM) supplemented with keratinocyte growth factor (KGF) and the Rho kinase inhibitor, Y-27632. The results demonstrated that SHEM medium supplemented with KGF and Y-27632 dramatically increased expression of corneal differentiation markers, keratin 3 and keratin 12, whereas expression of the progenitor marker, p63, did not appear to be significantly influenced by the choice of culture medium.

## 1. Introduction

Corneal epithelial progenitor cells are essential for maintenance of a smooth and transparent ocular surface. These so-called “corneal stem cells” are concentrated within the peripheral or limbal margin of the cornea [1] and express molecular markers typical of epithelial progenitor cells including the transcription factor p63 [2–4]. In contrast, mature corneal epithelial cells that cover the central cornea and suprabasal layers of the limbal epithelium are defined by coexpression of keratins 3 and 12 [1, 5–7].

Loss of corneal epithelial progenitor cells through disease or injury leads to a painful and blinding condition known as limbal stem cell deficiency (LSCD) [8–10]. Classic symptoms of LSCD include coverage of the corneal surface with conjunctival epithelial cells and vascularization of the corneal stroma. Strategies available for treating LSCD are based upon implantation of corneal epithelial progenitor cells derived from either an autologous or donor tissue source [11, 12]. Commonly, the epithelial progenitor cells are cultivated and implanted while attached to some form of scaffold. The most popular choice of scaffold for cultivated corneal epithelial implants is denuded amniotic membrane (AM). Limitations associated with the use of AM, however, including poor transparency and risk of disease transmission [13], have encouraged the development of alternative scaffolds such as membranes derived from the silk structural protein, fibroin [14].

In recent years, scaffolds fabricated from silk fibroin produced by the domesticated silkworm moth* Bombyx mori* (BMSF) have demonstrated potential for ophthalmic use due to their unique combination of tensile strength, controlled biodegradability, transparency, and compatibility with a range of ocular cell types [14–19]. For example, BMSF-coated surfaces and freestanding membranes support the attachment, growth, and differentiation of human corneal epithelial progenitor cells [14, 15, 20]. In these prior studies, epithelial cell attachment to BMSF has been facilitated through the use of serum-supplemented culture medium. Further improvements in corneal epithelial cell attachment and growth, however, may yet be achieved by coating the BMSF with purified extracellular matrix (ECM) proteins. Moreover, comparative studies of culture media for corneal epithelial progenitor cell growth on BMSF are lacking. In particular, there is mounting evidence that increased self-renewal and differentiation of cultured stem cells from ocular tissues can be achieved through the addition of the Rho kinase inhibitor, Y-27632, and keratinocyte growth factor (KGF) to culture medium [21–23]. The purpose of the present study was therefore to determine whether current protocols for the* ex vivo* expansion of human limbal epithelial (HLE) cell cultures on BMSF films could be improved by either (1) coating fibroin with specific ECM proteins or (2) utilising medium supplemented with Y-27632 and KGF. Previous investigations conducted on the use of BMSF-based scaffolds to support corneal epithelial cultures have used both immortalised and primary corneal epithelial cells [14, 15, 17, 24–26]. For comparative purposes, in the first part of this study, the effect of coating BMSF with ECM was therefore assessed using both an immortalised corneal epithelial cell line (HCE-T) and primary HLE cells. Cultures were assessed for cell adhesion, cell morphology, and culture confluence. In the second part of this study, the effect of different culture media on the expression of markers associated with progenitor or differentiated phenotypes was assessed in primary HLE cell cultures established on fibroin films.

## 2. Materials and Methods

### 2.1. Preparation of BMSF Films

*B. mori* cocoons with the pupa removed were supplied by Tajima Shoji Co. Ltd. (Yokohama, Japan). Aqueous solutions of silk fibroin (4% w/v) prepared from* B. mori* cocoons were processed using materials and techniques described previously and used within 1 month [14, 15]. Fibroin solution was poured into 24- or 96-well or T25 flasks to cast fibroin coatings. To assist drying, fibroin coatings were placed at room temperature (RT) in a fan-driven oven for 12 h and then water annealed (with a beaker of water) at −80 kPa in a vacuum chamber for 6 h at RT. Fibroin surfaces were sterilised in 70% ethanol for 30 min and then washed three times in PBS.

### 2.2. Coating of BMSF with ECM

BMSF-coated 24- and 96-well tissue culture plates were coated with ECM proteins at 5 *μ*g/mL to promote corneal reepithelialisation. Collagen I (Cell Matrix), collagen III (Millipore), collagen IV (Sigma-Aldrich), fibronectin and laminin (both from Sigma-Aldrich), and vitronectin (Promega) were diluted to 5 *μ*g/mL in PBS, applied to fibroin surfaces, and then dried at RT in a fan-driven oven for 12 h.

### 2.3. Establishment of HCE-T and Primary HLE Cells

#### 2.3.1. HCE-T Cells

Investigations were initiated using an immortalised human corneal epithelial cell line (HCE-T). Cells were propagated in DMEM High Glucose supplemented with 10% foetal bovine serum (FBS), 400 *μ*M L-glutamine, 100 IU/mL penicillin (Invitrogen), and 100 *µ*g/mL streptomycin (Invitrogen). An independent STR profile analysis of our working stocks by the Garvan Institute of Medical Research (Sydney, Australia) revealed 97% match with two reference HCE-T cell lines (RCB1384 and RCB2280).

#### 2.3.2. HLE Cells

After obtaining approval from the human research ethics committee (HREC) and donor consent from the Queensland Eye Bank (Brisbane, Australia), cadaveric human eye tissue was harvested to establish primary cultures of HLE cells. Limbal epithelial cells were removed by mechanical dissection (scraping with curved watchmaker forceps) following digestion of cornea for 1 hour in a 2.5 mg/mL Dispase (Gibco) solution at 37°C. Dissociated HLE cells were then propagated in the presence of irradiated murine 3T3 feeder cells in HLE medium (also referred to as Green's medium) as described previously [15]. Briefly, the medium was composed of a 3 : 1 mixture of Dulbecco's Modified Eagle's Medium and Ham F12 medium (Invitrogen) with 10% FBS, 1 mM HEPES buffer (Invitrogen), 5 *µ*g/mL human recombinant insulin (Actrapid), 0.4 *µ*g/mL hydrocortisone (Pharmacia), 4 *µ*M L-glutamine (Invitrogen), 2 pM triiodothyronine (Sigma-Aldrich), 200 nM adenine (Sigma-Aldrich), 100 IU/mL penicillin (Invitrogen), 100 *µ*g/mL streptomycin (Invitrogen), 0.25 *µ*g/mL amphotericin B (Invitrogen), and 10 ng/mL human recombinant EGF (Sigma-Aldrich).

Upon achieving 80% confluency, HLE cells and irradiated murine 3T3 feeder cells were seeded into fibroin-coated T25 flasks and 24-well tissue culture plates at a density of 1 × 10^5^ cells/cm^2^ and 2 × 10^4^ cells/cm^2^, respectively. HLE cells were subsequently cultured in Green's or supplemental hormonal epithelial medium (SHEM), a combination of Dulbecco's Modified Eagle's Medium/Ham's F-12 nutrient mixture (DMEM/F12; Invitrogen; 1 : 1) containing 1.05 mM calcium supplemented with 5 *µ*g/mL crystalline bovine insulin (Sigma-Aldrich), 30 ng/mL cholera-toxin (Calbiochem), 2 ng/mL EGF, 0.5% dimethyl sulfoxide (DMSO, Sigma-Aldrich), 0.5 *µ*g/mL hydrocortisone, 5 ng/mL sodium selenite, and 5 *µ*g/mL apotransferrin and supplemented with 10% FBS. SHEM media were also supplemented with Y-27632 (10 *µ*M) and KGF (10 ng/mL) (both from Invitrogen). Growth medium was changed at days 3, 5, and 7 and every other day after day 7 until completion. At 7 or 14 days, HLE cells cultured in 24-well tissue culture plates were fixed using 4% formaldehyde and washed 3 times in PBS for immunoflourescence. Simultaneously, HLE cells cultured in BMSF-coated T25 flasks underwent RNA isolation procedures for subsequent qPCR analysis.

### 2.4. Cell Attachment Assay

HCE-T or HLE cells were seeded into fibroin-coated wells at a density of 10^5^ cells/cm^2^. To avoid the unintended inclusion of cell attachment factors from serum-supplemented media, HCE-T cells were suspended in keratinocyte serum-free medium (KSFM; Thermo Fisher Scientific) containing 0.09 mM calcium supplemented with 30 mg/mL bovine pituitary extract, 0.2 ng/mL EGF, and ampicillin/streptomycin. Plates were incubated at 37°C in 5% CO_2_ for 90 min, washed once in PBS, and then fixed using 4% formaldehyde. Numbers of adherent cells were determined by staining cultures with Hoechst dye (Invitrogen) in 100 mM HEPES buffer for 1 h at RT to visualise cell nuclei. Cells were then washed 3 times in distilled water, visualised via fluorescent microscopy, and photographed using a standardised matrix of five (10x) fields of view. Cell numbers were determined manually using ImageJ software. This process was repeated using primary HLE cells.

### 2.5. Cellular Eccentricity and Confluency

To examine changes in cellular eccentricity and confluency, 3 × 10^4^ HCE-T cells/cm^2^ were seeded into 96-well ECM-coated fibroin plates in DKSFM. Plates were incubated inside the IncuCyte ZOOM live cell analysis system (Essen Bioscience) for 72 hours with photographs captured at hourly intervals. Subsequently after creating a processing definition, a set of basic metrics were analysed using phase object analysis to determine changes in cellular eccentricity (ranges from 0 to 1 with a perfect circle having a value of 0) and confluency (the percentage of the image area that is occupied by cells).

### 2.6. Phenotypic Analysis of HLE Cultures Grown in BMSF-Coated Wells

To assess potential differences in the phenotype of cells cultured in two different media on BMSF-coated surfaces, two markers of corneal epithelial cell phenotype, p63 (progenitor marker) and keratin pair 3/12 (stratifying, differentiated cells), were examined. A polyclonal rabbit antibody to p63 (1 : 100 dilution, BioLegend) and a mouse monoclonal antibody to cytokeratin pair 3/12 (1 : 700 dilution, Millipore) were used to perform immunocytochemistry. Initially, HCE cells on BMSF-coated wells were blocked in 5% normal goat serum (NGS) in PBS containing 0.5% Triton X-100 for 30 min. Primary antibodies diluted in 1% NGS were subsequently applied at RT for 1 h. Wells were washed 3 times in PBS to remove unbound primary antibody before the secondary antibody (1 : 200 dilution, Alexa Fluor 488 anti-mouse or Alexa Fluor 594 anti-rabbit; Invitrogen) was applied at RT for 20 min.

### 2.7. RNA Isolation

HLE cells cultured on BMSF-coated T25 flasks were processed for RNA extraction using methods described previously [27]. Briefly, RNA was extracted immediately prior to the commencement of culture (day 0) and also at day 7 and day 14 of culture. Cultured cells were treated with 1 mL TRIzol reagent (Invitrogen) and then combined with chloroform (200 *μ*L/mL lysate) for 2 minutes at RT. Cellular lysates were centrifuged at 12,000 ×g for 15 minutes at 4°C. Isopropyl alcohol (0.5 mL/mL lysate) was added to the aqueous phase and incubated for 10 minutes at RT. After centrifugation at 12,000 ×g for 10 minutes at 4°C, the supernatant was discarded and the RNA pellet washed with 1 mL 75% ethanol. Subsequently, RNA pellets were centrifuged at 7,500 ×g for 5 minutes at 4°C and then resuspended with 30 *μ*L ultrapure distilled water and stored at −80°C. RNA content was estimated by spectrophotometric analysis using the ND-1000 (Thermo Scientific) spectrophotometer and A260/A280 and A260/A230 ratios calculated to estimate RNA purity.

### 2.8. Reverse Transcription (RT)

RT was performed by mixing 2 *μ*g sample RNA, 1 *μ*L dNTPs (125 nM), 1 *μ*L random primers (pd(N)_6_; both from Promega), and ultrapure distilled water up to a volume of 10 *μ*L. Samples were incubated for 5 minutes at 65°C. Subsequently, 1 *μ*L Superscript III (200 U/*μ*L), 1 *μ*L RNAase OUT, 2 *μ*L 5x first-strand RXN buffer, 4 *μ*L MgCl_2_ (25 mM), and 2 *μ*L DTT (0.1 M, all from Invitrogen) were added to samples and then incubated for 10 minutes at 25°C, 1 hour at 55°C, and then 15 minutes at 70°C. cDNA samples were stored at −20°C.

### 2.9. Real-Time Polymerase Chain Reaction (qPCR)

Real-time PCR analysis was performed by combining 2 *μ*L sample cDNA (1 : 10 dilution) with 2 *μ*L forward primer and 2 *μ*L reverse primer (both 20 *µ*M, GeneWorks), 10 *μ*L SYBR green I (Roche), and 4 *μ*L ultrapure distilled water. A list of primers used in this study is shown in Table 1. The PCR reaction was initiated by incubation at 95°C for 10 minutes. This was followed by 45 cycles of (1) 95°C for 10 seconds, (2) 62°C for 10 seconds, and (3) 72°C for 10 seconds. Human beta-actin was employed as the reference gene while samples without reverse transcriptase or cDNA template were used as negative controls.

### 2.10. Statistical Analysis

The method of statistical analysis was two-way ANOVA. Significance level was set at *p* < 0.05 and* post hoc* testing between treatment means was conducted using Tukey's test.

## 3. Results

### 3.1. Adhesion of HCE-T and HLE Cells to ECM-Coated BMSF Films

**Figure 1:**
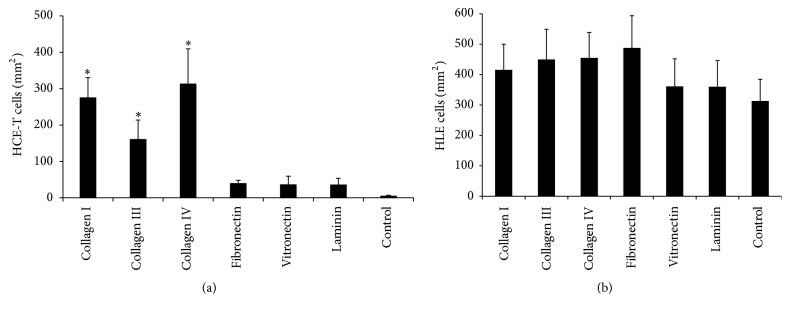
Adhesion of transformed HCE-T (a) or primary HLE (b) cells to BMSF coated with ECM proteins at 5 *μ*g/mL. Values represent mean ± SEM (HCE-T: *n* = 4; HLE: *n* = 5). For each cell type, an asterisk indicates that the value is significantly different (*p* < 0.05) from that obtained using uncoated (control) BMSF.

As shown in Figure 1(a), coating of BMSF films with collagen I, collagen III, or collagen IV significantly (*p* < 0.05) increased the number of adherent HCE-T cells. Increased HCE-T cell adhesion was also observed in response to coating with vitronectin, fibronectin, or laminin; however, none of these increases were significantly higher (*p* > 0.05) than the values obtained using uncoated fibroin.

In contrast to results obtained using HCE-T cells, ECM coating had no significant effect on the adhesion of primary HLE cells to BMSF films (Figure 1(b)). Furthermore, HLE cells exhibited a far higher tendency to adhere to uncoated fibroin than HCE-T cells. Specifically, the number of HLE cells that adhered to uncoated fibroin was approximately sixtyfold higher than that obtained using HCE-T cells (Figures 1(a) and 1(b)).

### 3.2. Morphology and Confluence of HCE-T and HLE Cells on ECM-Coated BMSF Films

**Figure 2:**
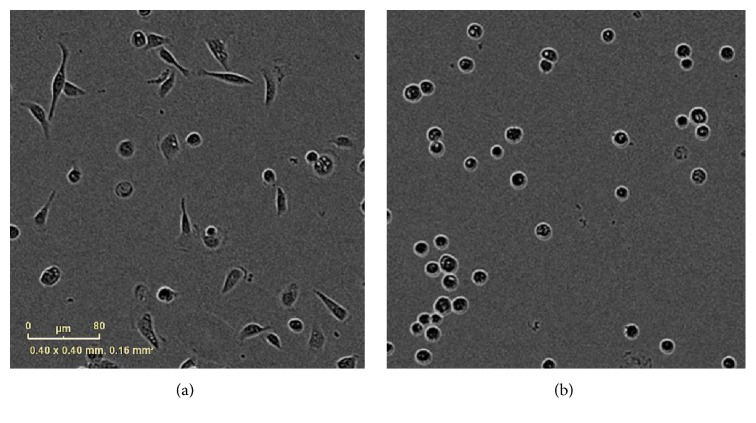
Morphology of HCE-T cells cultured for 1 hr on fibroin coated with collagen IV at 5 *μ*g/mL (a) or uncoated (control) fibroin (b). Scale bar = 80 *µ*M.

**Figure 3:**
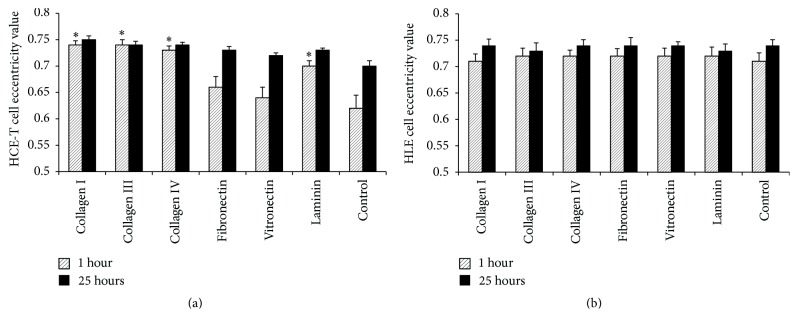
Eccentricity of transformed HCE-T (a) or primary HLE (b) cells cultured for 1 hour or 25 hours on BMSF films coated with ECM proteins at 5 *μ*g/mL. Values represent mean + SEM (HCE-T: *n* = 5; HLE: *n* = 3). An asterisk indicates that the value is significantly different (*p* < 0.05) from the value obtained using uncoated (control) BMSF.

 The morphology of HCE-T cells on ECM-coated BMSF films was examined after 1 hr and 25 hrs of culture. The cells on most films appeared relatively spherical; however, those on collagen and laminin coated films typically exhibited an elongated morphology with eccentricity values ranging from 0.7 to 0.75 (Figures 2(a) and 3(a)). After 25 hrs of culture, however, HCE-T cells on each of the coated BMSF films demonstrated a relatively elongated morphology with eccentricity values ranging from 0.7 to 0.75 (Figure 3(a)).

**Figure 4:**
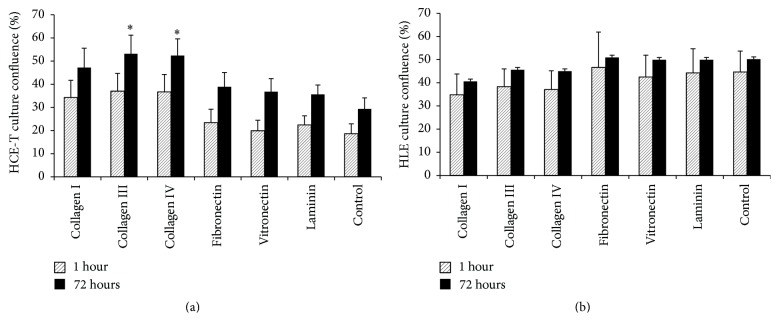
Confluency (%) of transformed HCE-T (a) or primary HLE (b) cell cultures after 1-hour or 72-hour growth on BMSF films pretreated with ECM proteins at 5 *μ*g/mL. Values represent mean + SEM (HCE-T: *n* = 4; HLE: *n* = 3). An asterisk indicates that the value is significantly different (*p* < 0.05) from that obtained using uncoated (control) BMSF.

In addition to the effect on cell morphology, coating of BMSF with collagen increased HCE-T culture confluence (Figure 4(a)). For example, by the end of the 72 hr culture period, HCE-T cultures established on fibroin coated with collagen III or collagen IV demonstrated almost double the level of confluence exhibited by those cultures established on uncoated fibroin.

In dramatic contrast to results obtained using HCE-T cells, none of the ECM coatings had a significant effect on HLE cell morphology or culture confluence at any point during the culture period (Figures 3(b) and 4(b)).

### 3.3. Influence of Culture Medium Characteristics on Gene Expression in HLE Cells

**Figure 5:**
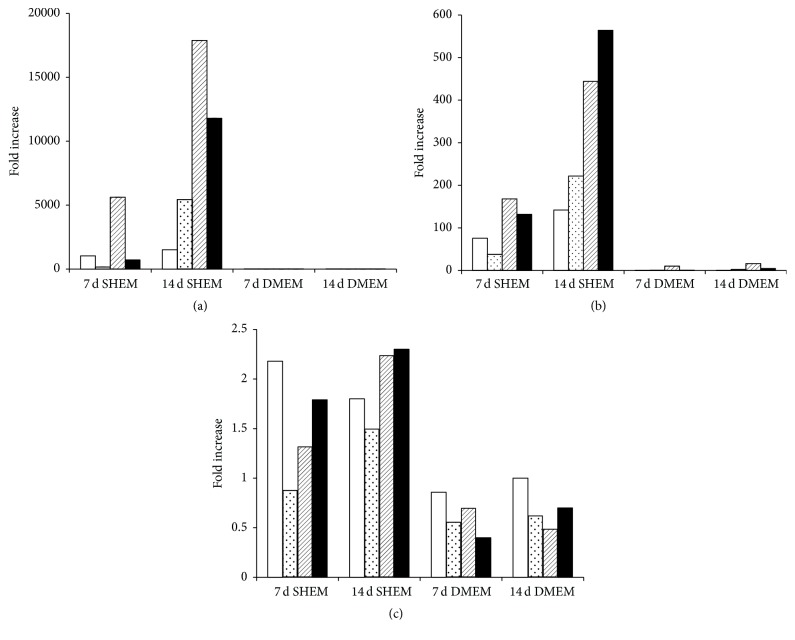
Fold increase in the expression of keratin 3 (a), keratin 12 (b), or p63 (c) in HLE cells from individual donors cultured in SHEM or DMEM for 7 or 14 days. Data from different treatment groups where cells were sourced from the same donor are indicated by identical fillings in individual bar graphs. Note: some values for cultures grown in DMEM in (a) and (b) are too low to register on the graph.

Messenger RNA transcripts for p63, keratin 3, and keratin 12 were quantified by real-time quantitative PCR in HLE cells grown on fibroin films in either SHEM medium (supplemented with Y-27632 and KGF) or DMEM for 0, 7, or 14 days. Results are presented in Table 2 and Figure 5. A consistent trend observed for all donors was that culture of HLE cells in SHEM medium supplemented with Y-27632 and KGF induced much greater relative increases in the expression of keratin 3 and keratin 12 over the culture period than was observed in response to culture with DMEM. For example, after 14 days, all HLE cultures grown in SHEM medium exhibited relative increases in keratin 3 expression that were at least 30-fold higher than those observed in donor matched controls cultured in DMEM (Figure 5(a)). Similar general trends were evident when the expression of keratin 12 in HLE cells cultured in SHEM was compared with data obtained from donor matched control groups cultured in DMEM (Figure 5(b)).

Evidence of p63 gene expression was detected in all HLE cultures, regardless of the donor tested, the length of the culture period, or the media used (Figure 5(c)). Overall, however, the fold change in p63 gene expression observed in response to culture with SHEM was relatively low (<2-fold) and not significantly different to that observed in donor matched groups cultured in DMEM. No significant evidence of p63 or keratin gene expression was detected in monocultures of 3T3 cells (data not shown).

### 3.4. Influence of Culture Medium on the Expression of Specific Protein Markers in HLE Cells Grown on BMSF Films

**Figure 6:**
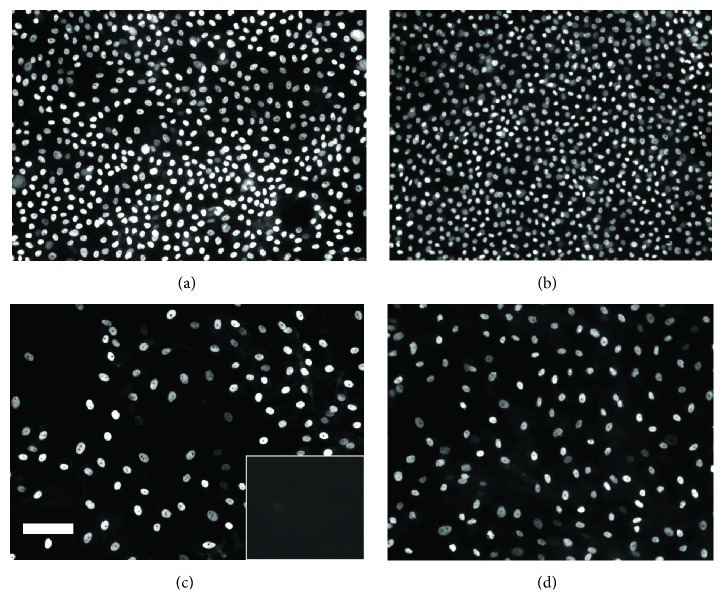
Fluorescent microscopy for p63 in HLE cells cultured on BMSF films in the presence of SHEM (a, b) or DMEM (c, d). Strong immunoreactivity (IR) for p63 was detected in all samples cultured for 7 d (a, c) or 14 d (b, d), regardless of the media used. No significant evidence of IR for p63 was detected in samples where primary antibody was omitted ((c), insert). Bar = 30 *µ*M.

**Figure 7:**
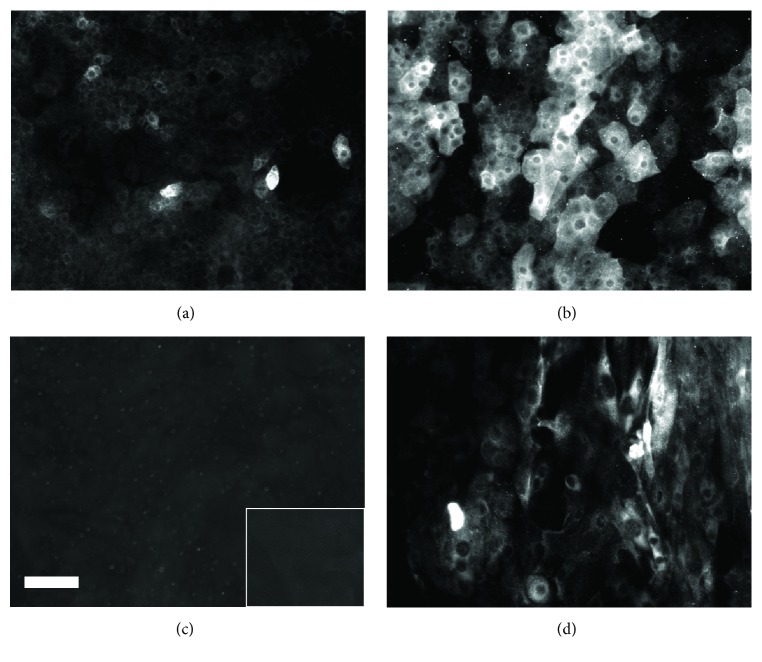
Fluorescent microscopy for keratin 3/12 in HLE cells cultured on BMSF films in the presence of SHEM (a, b) or DMEM (c, d). Keratin 3/12-IR was evident in a few sparsely scattered HLE cells in cultures grown in SHEM for 7 d (a). After 14 d, reactivity for keratin 3/12 was evident in all cultures regardless of the media used (b, d). No significant evidence of IR for keratin 3/12 was detected in samples cultured with DMEM for 7 d (c) or where primary antibody was omitted ((c), insert). Bar = 30 *µ*M.

HLE cells cultured on BMSF films in the presence of either SHEM or DMEM were investigated for the presence of p63 and keratin 3/12 by immunofluorescence at 7 and 14 days of growth. As shown in Figure 6, strong p63-IR was widely detected throughout all HLE cultures, regardless of the media used or the length of the culture period. In all instances, p63-IR was restricted to the cell nucleus. It was also noted that cells cultured in SHEM were generally smaller and present at a higher density than cells from donor matched groups cultured in DMEM. After 7 days of culture in SHEM, cytoplasmic K3/12-IR was detected in a few sparsely scattered cells throughout HLE cultures (Figure 7(a)). After 14 days of culture in SHEM, intense cytoplasmic K3/12-IR was detected throughout HLE cultures from all donors examined (Figure 7(b)). By contrast, no evidence of K3/12-IR was detectable at 7 days in donor matched groups cultured in DMEM (Figure 7(c)). K3/12-IR was detected at 14 days in cultures grown in DMEM; however, the extent and intensity of this fluorescence were less than those observed in donor matched groups cultured in SHEM (Figure 7(d)).

All negative controls (omission of primary antibody) for HLE cultures failed to demonstrate any significant fluorescence (Figures 6(c) and 7(c): inserts). In addition, no evidence of reactivity for either p63 or K3/12 was observed in monocultures of 3T3 cells (data not shown).

## 4. Discussion 

In previous investigations, we have shown that silk fibroin isolated from* B. mori* cocoons can be manufactured into flexible, permeable, and transparent scaffolds which support corneal epithelial cell attachment, growth, and differentiation [14, 15]. In the current study, we have attempted to optimize protocols for the expansion of corneal epithelial cells on BMSF* in vitro* by coating films with ECM proteins or including media supplements known to promote corneal differentiation and the retention of viable progenitor cell populations. In the first part of the study, it was shown that coating of fibroin films with ECM proteins, in particular collagen, significantly improved the adhesion and growth of immortalised corneal epithelial cells (HCE-T). These findings are in general agreement with those of other workers who have sought to improve protocols for the expansion of epithelial cell derived from the cornea. For example, it has been reported that coating tissue culture plastic with collagen or fibronectin significantly increases the expansion of human corneal endothelial cell cultures [18, 28–30]. Likewise, collagen or laminin coating enhances corneal epithelial cell attachment and migration* in vivo* and promotes increased stratification on polycarbonate membranes* in vitro* [31, 32]. Furthermore, human corneal epithelial cells demonstrate significantly higher rates of wound closure when cultured on therapeutic contact lenses coated with vitronectin [33]. Based on the evidence above and the response of HCE-T cells reported in the present study, we propose that coating BMSF-based scaffolds with ECM proteins may increase the potential of this biomaterial to promote increased epithelial cell adhesion and growth.

Although the finding that immortalised corneal epithelial cells demonstrate increased adhesion and growth on ECM-coated fibroin scaffolds is interesting from a scientific perspective, the potential value of this data for clinical applications is limited. In particular, the use of animal-based ECM proteins in the creation of tissue constructs for ocular surface repair or reconstruction may be problematic as it introduces potential risks associated with rejection or disease transmission. We have also demonstrated in the current study that there were major differences in the responses of immortalised and primary corneal cells to ECM-coated films. The HCE-T cell line was selected at the commencement of the current study as a convenient and robust model system to investigate options for improving protocols for the expansion of corneal cell cultures on BMSF-based scaffolds. This cell line has often been used as a model system by other workers to gain insights into corneal epithelial cell growth or function. In particular, the HCE-T cell line has been promoted as an alternative to the use of live animals for the study of corneal barrier function [34–36]. Nevertheless, HCE-T cells have a significantly altered genomic profile to primary corneal cells and this fact must be carefully considered when drawing conclusions on any data obtained through their use [37]. In the current study, the responses of HCE-T cells to coating fibroin films with ECM proteins were significantly different to those observed using primary HLE cells. Specifically, while HCE-T cells demonstrated increased cell attachment and growth in response to coating BMSF with ECM proteins, no such benefit was achieved for the culture of primary HLE cells. Furthermore, primary HLE cells demonstrated a much greater capacity to adhere to uncoated fibroin films than HCE-T cells and achieved much higher levels of confluence by the end of the culture period. Based on such data, we suggest that the HCE-T cell line will be only of limited value for any further attempts to optimize the expansion of HLE cells on BMSF-based scaffolds.

The second outcome of this study was that replacement of conventional keratinocyte growth media (DMEM) by medium supplemented with Y-27632 and KGF (SHEM) promoted a significant increase in corneal epithelial cell differentiation. This result is consistent with data obtained by other workers who have shown that supplementation of medium with Y-27632 and/or KGF improves epithelial cell growth and differentiation* in vivo* and* in vitro* [38]. For example, inclusion of Y-27632 in culture medium significantly increases in the long-term proliferative capacity of primary keratinocytes from a variety of anatomical locations [39]. Supplementation of culture medium with Y-27632 also promotes increased adhesion of dissociated HLE cells apparently by protecting these cells from the stress of reactive oxygen species [40]. There is strong evidence that KGF is also a potent inducer of epithelial cell division and differentiation including those epithelial cells in the cornea and limbus. For instance, corneal epithelial wound healing is enhanced by the topical application of KGF [38, 41] while expression of the KGF receptor, FGF2bR, is significantly upregulated in the mouse corneal epithelium after scrape injury [42]. The findings of the current study indicate that the use of Y-27632 and/or KGF is also beneficial for our attempts to promote corneal differentiation in HLE cultures established on BMSF-based scaffolds.

It is important to emphasise that, in this study, increased expression of genes associated with corneal differentiation was achieved without any apparent reduction in expression of the stem cell marker, p63. This outcome is highly significant since the long-term sustainability of any corneal epithelial construct requires the retention of a viable limbal progenitor population. Overall, the results of the current investigation compare favourably with those of other workers who have attempted to optimize protocols which sustain a progenitor population during HLE culture. For example, it has been shown that KGF secretion by limbal keratocytes* in vivo* promotes limbal epithelial cell growth and differentiation [43]. Furthermore, the action of KGF on limbal epithelial cells appears to be mediated by a pathway involving the expression of p63 [44]. Evidence has also been presented that KGF-supplemented media promote the outgrowth of limbal epithelial explant cultures on amniotic membrane (AM) which express significantly higher levels of p63 than in those exposed to HGF supplemented media [44]. It has also been shown that HLE cultures which contain cells exhibiting progenitor markers can be maintained for several months in the presence of SHEM supplemented with KGF and Y-27632 [23]. Based on such data, we recommend that further studies be conducted to determine whether the addition of Y-27632 and KGF to culture medium helps to promote the long-term viability of progenitor populations in HLE cell cultures established on BMSF-based scaffolds.

## 5. Conclusions

Based on our findings, we suggest that (1) the capacity of BMSF scaffolds to support the attachment or growth of some cell types (e.g., HCE-T) may be improved by coating with ECM proteins; however, this strategy offers no significant benefit for the culture of primary HLE cells; we also suggest that (2) the use of the SHEM medium supplemented with KGF and Y-27632 in our culture system promotes increased corneal differentiation without any apparent reduction in the expression of the progenitor marker, p63. This latter finding may be of benefit for future clinical studies designed to optimize protocols for the generation of HLE constructs on BMSF-based scaffolds for use in ocular surface reconstruction.

## Figures and Tables

**Table 1:** Oligonucleotide primers used for qPCR analysis.

Target		Sequence 5′ → 3′
Keratin 3	Forward primer	CCTCTACGACGCTGAGCTATC
	Reverse primer	CCAGGGAGCGATTATTGTCCAT

Keratin 12	Forward primer	CTCTCCTCGCAGAGTGTGATA
	Reverse primer	AACTAGAACCAAACATGGAAGC

p63	Forward primer	GTCATTTGATTCGAGTAGAGGGG
	Reverse primer	CTGGGGTGGCTCATAAGGT

Beta-actin	Forward primer	CATGTACGTTGCTATCCAGGC
	Reverse primer	CTCCTTAATGTCACGCACGAT

**Table 2:** Relative changes in gene expression in HLE cells in response to culture in different media (*n* = 4). An asterisk indicates that the value is significantly different (*p* < 0.05) from that obtained for the corresponding donor matched controls (DMEM).

Gene	Media	Days of culture	dCT	ddCT
p63	SHEM	7	5.95 + 0.28	−0.55 + 0.28
		14	5.3 + 0.16	−0.93 + 0.15
	DMEM	7	7.13 + 0.22	0.66 + 0.22
		14	6.97 + 0.21	0.5 + 0.21

Keratin 3	SHEM	7	6.72 + 1.15^*∗*^	−9.65 + 1.16^*∗*^
		14	3.67 + 0.82^*∗*^	−12.74 + 0.82^*∗*^
	DMEM	7	18.03 + 1.16	1.61 + 1.16
		14	14.52 + 0.29	−1.9 + 0.29

Keratin 12	SHEM	7	4.79 + 0.49^*∗*^	−6.46 + 0.49^*∗*^
		14	3.03 + 0.45^*∗*^	−8.2 + 0.45^*∗*^
	DMEM	7	12.02 + 1.46	0.77 + 1.46
		14	10.88 + 2.3	−0.36 + 2.3
